# Supplementary material for: Exploring the crosstalk between the FGF/FGFR pathway and tumor microenvironment in clear cell renal cell carcinoma
Source: PLoS One. 2026 Feb 10;21(2):e0339888. doi: 10.1371/journal.pone.0339888 (PMC12890122; doi:10.1371/journal.pone.0339888)
Supplement: Attachment — Submitted filename: Response to Reviewers.docx [file pone.0339888.s005.docx]

Pirkko L. Härkönen, MD, PhD

Academic Editor

*PLOS ONE*

15 December 2025

Dear Dr. Härkönen,

Re: PLOS ONE Decision: Revision required [PONE-D-25-37743] - [EMID:d440d231e20ceace]

Please find attached a revised version of our manuscript “Exploring the crosstalk between the FGF/FGFR pathway and tumor microenvironment in clear cell renal cell carcinoma”, which we would like to resubmit for consideration for publication as an Original Article in *PLOS ONE*.

Your comments and those of the reviewers were highly insightful and enabled us to greatly improve the quality of our manuscript. In the following pages are our point-by-point responses to each of the comments of the reviewers.

Revisions in the text are shown using yellow highlight. We hope that the revisions in the manuscript and our accompanying responses will be sufficient to make our manuscript suitable for publication in *PLOS ONE*.

We look forward to hearing from you at your earliest convenience.

Yours sincerely,

Takafumi Narisawa

Department of Urology, Faculty of Medicine, Yamagata University, Yamagata, Japan

Address: 2-2-2 Iida-Nishi, Yamagata City, Yamagata 990-9585, Japan

Email: tnari_0623@yahoo.co.jp

TEL: +81-23-628-5368

Reviewer #1

The fibroblast growth factor receptor (FGFR) family has garnered increasing attention in oncology due to its multifaceted role in GU tumors biology. This manuscript provides a timely and comprehensive investigation into the interplay between FGFR signaling and the tumor microenvironment in clear cell renal cell carcinoma (ccRCC). The study is well-conceived and methodologically sound, offering new insights into the biological significance of FGFR1 within the renal cancer microenvironment - an aspect that has not been fully elucidated in previous work. The data are clearly presented, the analyses are rigorous, and the conclusions are well supported by the findings. Overall, this is an elegant and well-executed study that meaningfully advances current understanding of FGFR biology in ccRCC. I recommend the manuscript for publication in its present form.

**Response to Reviewer:**

**We thank the Reviewer for the positive appraisal of our manuscript.**

Reviewer #2

Authors analyzed FGFR1-4 expression and CD163 positive cell count for estimation of TAM infiltration in 57 ccRCC specimens from patients undergoing nephrectomy using immunohistochemistry. This study indings indicate that FGFR1 could serve as a potential biomarker for therapeutic strategies and highlight the need for further research to explore FGFR-targeted therapies in ccRCC.

Authors described methods for quantification of IHC staining. “Staining of FGFRs was evaluated using a four-point scale (negative no staining: 0 point, weak stains to the same degree as: 1 point, moderate stains stronger: 2 point, strong stains considerably stronger: 3 point), and IHC scores were calculated as the mean point of three different areas per slide to account for intratumoral heterogeneity”

Q: However, it was not clear to me how they decide staining levels as “weak stains to the same degree as: 1 point, moderate stains stronger: 2 point, strong stains considerably stronger” for scoring? Please describe the method in more detail.

**Response to Reviewer:**

**Thank you for pointing this out. Two experienced physicians visually evaluated the staining intensity** **according to our previous study (Ref 11). We have added it in the Materials and Methods (Lines 112–115).**

Authors commented no availability of tumor samples from metastatic sites. However, it is critical to examine FGFR expression levels even using separate sets of clinical samples in this study.

C: Please show results of FGFR IHC in metastatic sites in addition to the primary tumors. FGFR4 showed trends of association with primary metastasis, and I wonder if FGFR4 expressions were selectively high in metastatic sites. Especially author showed association of high FGFR4 expression in RCC patients with metastases

**Response to Reviewer:**

**Thank you for your comments on this matter. The purpose of this study was to evaluate the FGFR expressions in primary tumor rather than in metastatic sites. Furthermore, the number of tumor samples from metastatic sites collected from the same patients in this study is very small. Therefore, in order to evaluate the molecular profile of metastatic lesions and its concordance with that of primary lesion, we have considered it is appropriate to conduct another study using a large number of paired samples and publish it as a separate study, which is ongoing in our institute. Since the lack of information on FGFR expressions in metastatic sites is one of the limitations of this study as you pointed out, we have added it in the Discussion (Lines 333–335).**

When applying a cut-off IHC score of 2.00, high expression levels were observed in 46 cases (80.7%) for FGFR1, 13 cases (22.8%) for FGFR2, and 8 cases (14.0%) for FGFR4 (Fig 1F)

Q: What is the rationale to use a cut-off IHC score of 2.00 for high vs low? There was no association of FGFR4 expression with survival, although there were trends of FGFR4 expression with synchronous metastasis and lymph node metastasis.

**Response to Reviewer:**

**Thank you for pointing this out. Since staining score of 2.00 means moderate, we determined IHC score of 2.00 as cut-off is suitable for dividing into high and low. Furthermore, when applying a cut-off IHC score of 2.00, the distribution of FGFR expressions was consistent with previous reports (Ref 6, 7, 11). We tried validating with several cut-off scores, but did not find any association of FGFR4 expression with survival in any of cut-off scores (cut-off = 1.0, hazard ratio = 1.27 [95%CI: 0.43–3.75]; cut-off = 1.2, hazard ratio = 1.33 [95%CI: 0.60–2.97]; cut-off = 1.5, hazard ratio = 1.70 [95%CI: 0.73–3.99]; cut-off = 2.0, hazard ratio = 1.00 [95%CI: 0.30–3.37]).** **Possible reason why FGFR4 expression and prognosis did not correlate is that treatment modality differed among patients as described in the Discussion (Lines 286–288).**

Analysis of FGFR subtype expression levels and intra-tumor macrophage infiltration

Q: Please confirm if intra-tumor macrophage expressed FGFR by co-staining with CD163.

**Response to Reviewer:**

**Thank you for your suggestion. Previous study (Ref 18) indicated tumor-associated macrophage express FGFRs and we would like to refer to it in the Introduction (Lines 82–83). Immunohistochemical analyses of FGFRs in this study were conducted under a time-limited collaborative research agreement with an industry partner (Eisai Co., Ltd.), who provided and validated the FGFR antibody panel and covered the costs of exploratory staining. This agreement has now expired, and under the terms of the agreement and our institutional policies, we are unfortunately unable to perform additional double-staining experiments on this cohort. However, to address your query regarding the spatial relationship, we present representative single-plex images below for your review (Reviewer Fig 1). These images illustrate FGFR1-positive tumor/stromal cells in perivascular regions where CD163-positive macrophages accumulate. We have decided not to include these single-plex images in the revised manuscript to avoid any potential confusion for readers, as these images demonstrate spatial proximity but do not definitively prove co-expression.**

**Reviewer Fig 1. Association between FGFR1-expressing tumor/stromal compartments and CD163-positive cells.**

Representative single-plex images illustrating FGFR1-positive tumor/stromal cells in perivascular regions where CD163-positive macrophages accumulate as shown by the arrow. (A) Representative image of FGFR1 staining. (B) Representative image of CD163 staining.
